# Assessing Child-to-Parent Violence With the Child-to-Parent Violence Questionnaire, Parents’ Version (CPV-Q-P): Factor Structure, Prevalence, and Reasons

**DOI:** 10.3389/fpsyg.2020.604956

**Published:** 2020-11-26

**Authors:** Lourdes Contreras, Samuel P. León, M. Carmen Cano-Lozano

**Affiliations:** ^1^Department of Psychology, University of Jaén, Jaén, Spain; ^2^Department of Education, University of Jaén, Jaén, Spain

**Keywords:** child-to-parent violence, parents, adolescents, assessment, prevalence

## Abstract

Child-to-parent violence has dramatically risen in the last decade, becoming a concerning issue in many countries, so research on this issue has also increased. However, most of the studies on this topic have been conducted with samples of adolescents, and very few with samples of parents. In addition, the variety of assessment instruments does not reflect the elements of this type of violence. Thus, the current study was aimed to examine the factor structure, reliability, and validity of the Child-to-parent Violence Questionnaire, parents’ version (CPV-Q-P), in a sample of Spanish parents of adolescents. Moreover, the prevalence rates of the different types of violence and the reasons for violence were also examined. A total of 1,012 Spanish parents of adolescents aged between 12 and 17 years old (55.1% mothers, 44.9% fathers) were assessed using the CPV-Q-P. Data indicated a matrix of four factors with 14 items, assessing psychological violence, physical violence, financial violence, and control/domain over parents, and two factors with 8 items capturing the reasons for child-to-parent violence (instrumental and reactive), with adequate psychometric properties. The more frequent type of violence was control and domain over parents, followed by psychological, financial, and physical violence, with no significant differences between mothers and fathers. Otherwise, instrumental reasons were more frequent than reactive types, with no differences between mothers and fathers. The CPV-Q-P is a useful instrument to assess child-to-parent violence from the parents’ perspective in both professional and research settings.

## Introduction

Child-to-parent violence (CPV) has dramatically increased in the last decade, becoming a concerning issue across different countries (e.g., Margolin and Baucom, 2014; Ibabe, 2016; Beckmann et al., 2017; Simmons et al., 2018; Contreras et al., 2020). This type of family violence is defined as those behaviors that are intended to cause psychological, physical, or financial damage to gain power and control (Cottrell, 2001) and to dominate parents (Howard and Rottem, 2008, p. 10; Molla-Esparza and Aroca-Montolío, 2018, p. 17). Some authors also indicate that, in CPV cases, it is necessary to exclude isolated acts of violence (Pereira et al., 2017; Molla-Esparza and Aroca-Montolío, 2018).

Regarding the different types of CPV according to Cottrell (2001), psychological violence refers to some behaviors such as intimidations and threats, among others, and also to verbal behaviors such as shouting, insulting, or challenging. Physical violence refers to acts such as pushing, kicking, or punching, and financial violence includes behaviors such as stealing money or parents’ belongings, demanding parents buy things they feel they cannot afford, or incurring debts the parents must cover. The control, domination, and power over parents are reflected in such behaviors as making unrealistic demands on parents (for example, insisting they drop what they are doing to comply with the child’s demands) or controlling the running of the household. These types of abuse can occur at the same time, and in fact, they overlap to a certain extent (Cottrell, 2001), resulting in an escalation of violence from psychological abuse to a more severe form of violence such as physical abuse (Cottrell, 2001; Eckstein, 2004). In addition, CPV behaviors can be reactive or instrumental (Calvete et al., 2015; Contreras et al., 2019, 2020). Reactive violence occurs in response to a previous provocation, real or perceived, whereas instrumental violence refers to the use of aggression to obtain something (Crick and Dodge, 1996).

In Spain, the Fiscalía General del Estado de España (2020), in its last report, expresses concern about the notable increase in CPV cases over the last decade (4,665 in 2017, 4,871 in 2018, and 5,055 in 2019). Nevertheless, as these data refer to those reported cases at Juvenile Court, it is expected that many cases of CPV remain unknown. In this regard, studies with community samples provide a relevant source of information about the extent of CPV. There are many field studies across countries in which adolescents report CPV incidents, but studies with samples of parents reporting their children’s violent behaviors are scarce. However, to know the parents’ perspectives about CPV is crucial for a more accurate understanding of this phenomenon (Contreras et al., 2019), as some discrepancies have been observed between adolescents’ reports and parents’ reports (Calvete et al., 2017; Ibabe, 2019) in the sense that parents may underestimate the violence they suffer from their children (Calvete et al., 2017). Most of these studies had been conducted with qualitative methods such as interviews or focus groups with parents (e.g., Jackson, 2003; Cottrell and Monk, 2004; Edenborough et al., 2008). The studies with quantitative methods and their assessment instruments are briefly described below.

Some authors have focused exclusively on child-to-mother violence, such as, for example, Edenborough et al. (2011), who developed the Child-to-Mother Scale (CMVS), which includes nine items measuring a unidimensional construct of CPV. The instrument also incorporated a second part exploring triggers of threatening and/or violent behaviors, but the authors did not report the prevalence rates of CPV. In this line, Abbaspour et al. (2019) recently developed and validated the Parent Abuse Scale (girl-mother). This scale is composed of 14 items describing physical and emotional violent behaviors, and the authors do not inform about prevalence rates of CPV in Iran in their study. Very recently, Simmons et al. (2019a) have designed the Abusive Behavior by Children-Indices (ABC-I), an instrument aimed to differentiate normative behavior towards parents from CPV with 10 behavior descriptors of physical aggression, verbal aggression, and coercive behavior (which includes financial and emotional abuse). In this study, 38% of parents described their child as abusive. However, as these items were derived from the Beliefs About Child-to-Parent Abuse Questionnaire (BACPAQ; Simmons et al., 2019b), a previous study on social norms about CPV in Australia, the authors recommend, in case of research outside this country, the administration of the BACPAQ together with the ABC-I to identify cultural-specific thresholds for abuse.

In the Spanish context, Calvete et al. (2017) assessed 880 parents of adolescents from the Basque Country with the parent’s version of the Child-to-Parent Aggression Questionnaire (CPAQ; Calvete et al., 2013). This instrument is composed of 10 items, from which seven describe psychological aggression and three describe physical aggression. More recently, Ibabe (2019) evaluated a sample of 161 pairs of parents (mothers and fathers) of adolescents aged 12–18 with a version of the Conflict Tactics Scale Child-Parents (CTS1, Straus et al., 1998). This scale contains 13 items to assess psychological and physical violence. Regarding the prevalence rates in Spain, when CPV is evaluated considering the presence of violent acts at least in one occasion in the last year, psychological violence oscillates between 81.9 and 88% towards the mother, and between 75.7 and 82% towards the father. Physical violence oscillates between 2.3 and 10.9% and between 1.9 and 6.9% towards the mother and the father, respectively (Calvete et al., 2017; Ibabe, 2019). When prevalence is estimated assessing reiterated violence, results show 6.4 and 4.8% of psychological violence towards the mother and father, respectively, as well as 2.8 and 1.2% of physical violence towards the mother and father, respectively (Calvete et al., 2017).

The study of a complex phenomenon such as CPV requires the assessment of different sources of information (perpetrator and victim), as it is important to explore their perceptions of the problem. As reflected, the available instruments to assess CPV from the parents’ perspectives are very scarce and they reflect the variability and inconsistency in the conceptualization of this phenomenon in each study. Some of these instruments assess only some types of CPV, such as psychological and physical violence towards parents (Calvete et al., 2017; Ibabe, 2019) or emotional and physical violence (Abbaspour et al., 2019). Otherwise, some scales are focused exclusively in child-to-mother violence (Edenborough et al., 2011; Abbaspour et al., 2019). Thereby, we intend to develop and validate an instrument that assesses a wide range of CPV behaviors from the parents’ perspective, including psychological, physical, and financial violence (Cottrell, 2001), and also control (Cottrell, 2001) and domain over parents (Howard and Rottem, 2008; Molla-Esparza and Aroca-Montolío, 2018), as this is a crucial component of CPV. In fact, in CPV cases, there is an inversion of conventional power relations within the family, changing the traditional and expected parents-children power relation into a relation in which children have the power over parents (Tew and Nixon, 2010). In this regard, although the ABC-I (Simmons et al., 2019a) incorporates a coercive component, it refers to financial violence (e.g., “Stole money or possessions from parents”) and emotional abuse (e.g., “Attempted to intimidate a parent”). Very recently, Contreras et al. (2019) have developed and validated the Child-to-parent Violence Questionnaire, adolescent’s version (CPV-Q) with good psychometric properties. This instrument consists of 14 parallel items measuring different acts of CPV (psychological, physical, and financial violence, and control/domain over parents) and also includes eight reasons for the aggressions against parents. Its structure has been also replicated with other samples of adolescents from other countries (e.g., Jiménez-García et al., 2020). Consequently, the main purpose of the current study is to examine the structure, reliability, and validity of the Child-to-parent Violence Questionnaire, parents’ version (CPV-Q-PV) in a sample of Spanish parents of adolescents. The CPV-Q-P includes the same violent behaviors towards parents as the adolescents’ version. In addition, this study is also aimed to explore the prevalence rates of the different types of CPV and the reasons for the violence, from the parents’ perspectives.

## Materials and Methods

### Sample

The sample consisted of 1,012 parents of adolescents aged between 12 and 17 years old (55.1% mothers, *M*_age_ = 46,19, *SD* = 6.27; 44.9% fathers, *M*_age_ = 48.34, *SD* = 6.27), from Andalucía (Southern Spain). The 85% of parents were married, 8.8% were divorced or separated, and 3.6% were living together but not married.

We calculated the minimal sample size at 95% confidence level, with a 5% confidence interval at 80% of statistical power. In this regard, the estimated minimum sample size was 385. Following Hair et al. (2010), the general rule to estimate the minimum sample size to perform factor treatment in a survey implies to have a minimum of five observations per variable (5:1). In our study, as the scale consisted of 22 items, the minimum sample size for the factorial treatment would be 111.

### Instruments

#### The Child-to-Parent Violence Questionnaire, Parents’ Version

It comprises a total of 14 items (as in the adolescents’ version) referring to different acts of psychological (four items), physical (three items), and financial violence (three items), and also behaviors demonstrating control and domain over parents (four items) (see Appendix). In this version, parents are asked to indicate how often their children have showed each of the behaviors against them in the past year, with a five-point scale of frequency: 0 (never), 1 (rarely = it has occurred once), 2 (sometimes = 2–3 times), 3 (many times = 4–5 times), and 4 (very often = more than 6 times). It also includes eight reasons for the aggressions against parents, instrumental (five items) and reactive (three items), also using a four-point scale: 0 (never), 1 (sometimes), 2 (almost always), and 3 (always). This second part of the instrument is completed if participants respond positively to the items of the aggressions.

#### The Warmth Scale (WS), Parents’ Version

The WS (Fuentes et al., 1999) consists of 20 items, with two factors referring to the support dimension of the parenting style: Affection/Communication and Criticism/rejection by parents towards their children. Each factor includes 10 items with a scale ranging from 1 (never) to 5 (always). In this study, Cronbach’s alpha was 0.90 for the Affection subscale and 0.85 for Criticism/rejection subscale.

### Procedure

We obtained authorization from the Ethics Committee of the University of Jaén (Spain) (reference OCT.19/1.PRY). The sample was firstly obtained through contact with different high schools, offering to parents of adolescents aged 12–17 years to participate in this study. Then, the sample was completed with snowball sampling. This is a process where initial informants are recruited and then are asked to use their networks to recruit additional participants (Jackson et al., 2003). Participants received and signed the informed consent previously to the assessment, and each participant received an identification code to guarantee the confidentiality of the data. The study was conducted with PAPI (Paper-and-Pencil Interviewing). No incentive was offered in exchange for participation, and the evaluations were conducted individually.

### Data Analysis

The R software was used to conduct all analyses. The *α* value for all statistical tests was set to 0.05. Data screening was performed before doing the factorial analysis to evaluate the distribution of data and assumptions. For missing values, treatment multiple imputation was made with the MICE package of R (Buuren and Groothuis-Oudshoorn, 2011). The lavaan R package (Rosseel, 2012) was used to conduct confirmatory factorial analysis (CFA). Robust maximum likelihood (MLR) with robust standard errors and a scaled test statistic was used as estimation method for CFA (Finney and DiStefano, 2013) to account for multivariate non-normality. The estimation errors resulting from CFA that shared the same latent variable with a Modification Index (IM) greater than 10.83 (*α* = 0.001) were covariates (Hermida, 2015). Cronbach’s *α* and McDonald’s *ω* were used to measure the reliability of the scale. Furthermore, following Carretero-Dios and Pérez (2007), the correlations between each dimension of the CPV-Q-P and the dimensions of the WS (Fuentes et al., 1999) were used to search for external evidence of validity (convergent validity), as previous studies have found that CPV is related both to lower levels of affection/communication and to higher levels of Criticism/rejection from parents (Gámez-Guadix et al., 2012; Contreras and Cano-Lozano, 2014).

Otherwise, the percentages of the types of CPV (psychological, physical, financial, and control/domain) towards the mother and the father were calculated. Differences between fathers and mothers were examined through the chi square statistic, analyzing the effect size with the *V* Cramer coefficient. In this regard, we first explored the presence of any type of CPV behavior, at least in one occasion in the last year (any answer different from 0 in the response scale), which provides a general perspective of the more frequent CPV behaviors. In addition, in order to obtain a more relevant indicator, we also estimated the presence of CPV considering the percentage of parents who reported having received those violent behaviors repeatedly in the last year (response 2 or higher in the Likert scale), for each type of CPV. Besides, to explore the mean differences between fathers and mothers regarding the reasons for CPV, *t*-test for independent samples was carried out, calculating the effect size through eta square statistic. Finally, the invariance of the model proposed for the parents’ gender at the configural, metric, scalar, and strict level was analyzed.

## Results

Before the factorial treatment of the scale, it was necessary to evaluate the previous assumptions to verify that the data could be treated by this type of analysis. For additivity, we tested the correlations between the items. No item showed multicollinearity (*r* > 0.90) or singularity (*r* > 0.95). A linear regression was generated with random numbers and scale scores to evaluate the assumptions of linearity, homogeneity, and homoscedasticity. The distribution of the residues resulting from the regression was evaluated. The resulting distribution was not violating any assumptions, showing a distribution of standardized regression residuals mostly between −2 and +2.

### Confirmatory Factor Analysis

The estimator used for the CFA was MLR, as our data did not show multivariate normality (Maximum Likelihood estimation with Robust, Hardin and Hilbe, 2012. The results showed a good fit of the model (Hair et al., 2010), *χ*^2^ (189) = 561.95, *p* < 0.001, CFI = 0.918, TLI = 0.899, SRMR = 0.053, RMSEA = 0.044 (RMSEA 90% CI [0.041, 0.047]), AIC = 55,512, and BIC = 55,827. The reliability analysis resulted in *α* = 0.755, *ω* = 0.779, indicating that the scale showed acceptable reliability. Table 1 shows the factor loading and internal consistency of the factors. All the covariation relationships between variables were significant (see Table 2).

**Table 1:** Factor loading and internal consistency of the factors of the Child-to-parent Violence Questionnaire, Parents’ version (CPV-Q-P).

Item	I	II	III	IV	IR	RR
***CPV behaviors***						
1	0.68					
2	0.65					
3	0.69					
4	0.69					
8		0.84				
10		0.74				
11		0.63				
6			0.64			
7			0.71			
12			0.68			
5				0.41		
9				0.64		
13				0.83		
14				0.62		
***Reasons for CPV***						
1					0.66	
2					0.79	
3					0.75	
4					0.60	
5					0.50	
6						0.54
7						0.49
8						0.54
Cronbach’s *α*	0.80	0.77	0.54	0.67	0.77	0.52
McDonald’s *ω*	0.81	0.82	0.68	0.70	0.78	0.62

CPV, child-to-parent violence; I: psychological; II: physical; III: financial; IV: control/domain; IR, instrumental reasons; RR, reactive reasons.

**Table 2:** Factor covariances for latent variables.

		Estimate	SE	95% CI		*Z*	*p*	Standard Estimate
				Lower	Upper			
Psychological	Phy	0.71	0.028	0.66	0.77	25.2	<0.001	0.71
	Fin	0.79	0.027	0.73	0.84	28.5	<0.001	0.79
	C.D	0.73	0.027	0.67	0.78	26.4	<0.001	0.73
Physical	Fin	0.71	0.028	0.65	0.76	25.3	<0.001	0.71
	C.D	0.62	0.029	0.56	0.68	21.4	<0.001	0.62
Financial	C.D	0.63	0.030	0.57	0.69	21.1	<0.001	0.63
IR	RR	0.77	0.043	0.69	0.86	17.9	<0.001	0.77
CPV	Psy	1.34	0.369	0.61	2.56	3.64	<0.001	0.93
	Phy	0.60	0.145	0.31	1.23	4.14	<0.001	0.75
	Fin	0.90	0.195	0.51	1.91	4.61	<0.001	0.86
	C.D	0.72	0.098	0.52	1.76	7.34	<0.001	0.80
CPV	IR	0.80	0.030	0.74	0.86	26.93	<0.001	0.48
	RR	0.78	0.062	0.66	0.91	12.67	<0.001	0.42

Psy, psychological; Phy, physical; Fin, financial; C.D, control/domain; CPV, child-to-parent violence; IR, instrumental reasons; RR, reactive reasons.

### Parental Gender Invariance

The dimensionality of the model was explored with the analysis of the invariance for the parents’ gender. This analysis was aimed to assess if the dimensionality of the model was equivalent for the mother and the father. Table 3 shows the results of the analysis of invariance for configural, metric, scalar, and strict levels. As shown, all the levels of invariance were reached, as the changes from one level to another level were not different more than 0.01 in CFI, together with the changes of RMSEA higher than 0.015 with respect to the more restrictive model (Chen, 2007).

**Table 3:** Fit indices for parental gender invariance.

	Chi	*df*	*p*	CFI	ΔCFI	RMSEA	RMSEA (CI 90%)	ΔRMSEA
Configural	809.82	378	<0.01	0.913	-	0.062	0.056–0.068	-
Metric	772.70	390	<0.01	0.917	0.004	0.060	0.053–0.066	−0.002
Scalar	806.44	412	<0.01	0.917	0.000	0.058	0.052–0.064	−0.002
Strict	784.25	434	<0.01	0.920	0.003	0.055	0.049–0.062	−0.002

df, degree of freedom; CFI, comparative fit index; RMSEA, root mean square error approximation; ΔCFI, comparative fit index increase; CI, confidence interval; ΔRMSEA, root mean square error approximation increase.

### Evidence of Convergent Validity

The correlations between the dimensions of the CPV-Q-P and the dimensions of the Warmth Scale (Affection/Communication and Criticism/rejection) were all statistically significant (*p* < 0.001). Concretely, CPV dimensions were related to lower levels of affection/communication and to higher levels of Criticism/rejection (see Table 4).

**Table 4:** Bivariate correlations between the dimensions of the Child-to-parent Violence Questionnaire, Parents’ version (CPV-Q-P) and the dimensions of Warmth Scale.

	Psychological	Physical	Financial	Control/domain	Instrumental reasons	Reactive reasons
Affection	−0.241	−0.200	−0.287	−0.266	−0.323	−0.243
Criticism	0.312	0.255	0.303	0.317	0.332	0.328

All correlations were significant at the *p* < 0.001 level.

### Prevalence of CPV

Table 5 shows the percentages of types of CPV towards mothers and fathers. The more frequent type of CPV was control/domain, followed by psychological, financial, and physical violence. With respect to the differences according to the victims’ gender, although mothers reported higher frequencies in CPV behaviors in comparison to fathers, results indicated no statistically significant differences between mothers and fathers in the proportion of any type of violence. Regarding the reasons for CPV, instrumental reasons were more frequent than reactive reasons, with no significant differences between mothers and fathers (see Table 5).

**Table 5:** Percentages of CPV and reasons (means) for CPV. Differences among father and mother.

Types of CPV	Total *N* = 1,012 (%)	Mother *n* = 558 (%)	Father *n* = 454 (%)	χ^2^	*V*
***At least in one occasion***					
Psychological	45.50	25.70	19.80	0.65	0.02
Physical	7.40	4.20	3.20	0.15	0.01
Financial	33.60	18.30	15.30	0.11	0.01
Control/domain	78.60	43.60	35.00	0.16	0.01
***Reiterated violence***					
Psychological	18.60	11.20	7.40	2.30	0.05
Physical	2.60	1.70	0.90	1.13	0.03
Financial	13.60	6.80	6.80	1.70	0.04
Control/domain	52.40	29.30	23.00	0.36	0.02
**Reasons for CPV**	**Total *M* (*SD*)**	**Mother *M* (*SD*)**	**Father *M* (*SD*)**	***t***	***η*^2^**
Instrumental	0.47 (0.51)	0.44 (0.49)	0.51 (0.53)	1.89	0.00
Reactive	0.41 (0.46)	0.40 (0.44)	0.42 (0.48)	0.736	0.00

CPV, child-to-parent violence.

## Discussion

The main objective of the current study was to analyze the factor structure, reliability, and validity of the CPV-Q-P in a sample of Spanish parents of adolescents. In addition, this study was also aimed to explore the prevalence rates of the different types of CPV and the reasons for the violence from the parents’ perspectives. The CFA indicated that the CPV-Q-P shows a structure with four factors (psychological violence, physical violence, financial violence, and control/domain), with adequate psychometric properties. The CPV-Q-P also includes eight reasons for CPV, grouped into two factors (reactive and instrumental reasons), also with adequate psychometric properties. Consequently, the structure obtained for the CPV-Q-P is similar to the adolescents’ version (CPV-Q, Contreras et al., 2019). Regarding the provision of convergent validity, the results indicate that the CPV behaviors are related to lower levels of affection/communication and to higher levels of Criticism/rejection from parents, in line with previous studies (Gámez-Guadix et al., 2012; Contreras and Cano-Lozano, 2014).

With regard to the prevalence rates, results showed that the more frequent type of CPV was control/domain, followed by psychological, financial, and physical violence. In respect of the differences according to the victim’s gender, although mothers reported higher frequencies in all the CPV behaviors in comparison to fathers, data indicated no statistically significant differences between fathers and mothers in the proportion of any type of violence. Similarly, other studies also show higher frequencies of violence towards the mother than the father (Calvete et al., 2017; Ibabe, 2019). When CPV is evaluated considering the presence of violent acts at least in one occasion in the last year, our percentages are lower than those found in previous studies in the Spanish context (Calvete et al., 2017; Ibabe, 2019). One explanation could be that in the questionnaire used in the study by Calvete et al. (2017) (the CPAQ; Calvete et al., 2013), it included the item “You have shouted at your parents when you were angry” to evaluate psychological violence, whereas this behavior is not evaluated in the CPV-Q-P. This item refers to a very frequent behavior in adolescents in their relationships with their parents during this life period, so it is likely that most of the adolescents inform having shouted at their parents at least once during the last year. This could have caused the high percentages of this type of CPV in previous studies. Otherwise, percentages of physical violence towards the mother and the father are in line with previous results (Calvete et al., 2017; Ibabe, 2019).

Estimating the prevalence assessing reiterated violent acts gives us a more accurate picture of the real cases of CPV. In fact, as adolescence is usually a time of tension between parental authority and adolescent’s increasing need for autonomy, it is necessary to mark a clear boundary between CPV and problematic behaviors that could be regarded as “usual” adolescent behavior (Coogan, 2011). When CPV is evaluated in this way, percentages of psychological and physical violence are more similar to previous literature (Calvete et al., 2017). In respect of financial violence, previous studies with parents have not reported data on this type of violence, so we cannot compare our results. Otherwise, our study reveals that control and domain over parents are the more frequent types of CPV. In this regard, “such misuse of power by the child clearly distinguishes CPV from the kind of behaviors that may be regarded as part of conventional journey through developmental stages” (Coogan, 2014, p. 4). However, as no previous researches have explored this particular form of CPV, it is not possible to compare our data about control and domain over parents with previous literature. Finally, with respect to the reasons for CPV, parents reported instrumental reasons with higher frequency than reactive reasons, with no differences between mothers and fathers.

Notwithstanding, this study presents some limitations that must be considered. First, these data refer to a wide sample of Spanish parents of adolescents that belong to a particular cultural and social context, so this aspect must be considered when generalizing the results. Second, future studies should provide, for example, the test–retest reliability of the scale. Despite these limitations, the results indicate that the CPV-Q-P is a valid instrument for assessing a wide variety of CPV behaviors from the parents’ perspective, together with the reasons for the violence. As aforementioned, exploring the perspectives of the actors involved in CPV (parents and children) is basic, as they might have different perceptions of the problem. This fact has clear implications. Regarding the research field, having both sources of information gives us a more accurate picture of the reality of this phenomenon. In respect of the professional context, knowing both perceptions of the problem will facilitate the design of specific treatment program for families immersed in this type of violence, in which the intervention with both children and parents is crucial. Finally, now that we have a validated instrument to assess CPV, with two parallel versions (adolescents and parents), in future studies, we will investigate this type of violence with samples of adolescents and parents together, with the aim to conduct an integral evaluation of this form of family violence.

## Data Availability Statement

The raw data supporting the conclusions of this article will be made available by the authors, without undue reservation.

## Ethics Statement

The studies involving human participants were reviewed and approved by Ethics Committee of the University of Jaén (Spain; reference OCT.19/1.PRY). The patients/participants provided their written informed consent to participate in this study.

## Author Contributions

LC and MC-L: conceptualization, methodology, writing — review, editing, and funding acquisition. SL: validation, formal analysis, and data curation. LC, MC-L, and SL: writing — original draft preparation. LC: project administration.

### Conflict of Interest

The authors declare that the research was conducted in the absence of any commercial or financial relationships that could be construed as a potential conflict of interest.
