# Supplementary material for: Assessing Child-to-Parent Violence With the Child-to-Parent Violence Questionnaire, Parents’ Version (CPV-Q-P): Factor Structure, Prevalence, and Reasons
Source: Front Psychol. 2020 Nov 26;11:604956. doi: 10.3389/fpsyg.2020.604956 (PMC7725712; doi:10.3389/fpsyg.2020.604956)
Supplement: Supplementary file 1 [file Data_Sheet_1.pdf]

Appendix.

Child-to-Parent Violence Questionnaire Parents' Version (CPV-Q-P) (English)

Please indicate how often your son/daughter has carried out each of the following behaviours against you in the past year.

0 = Never   1 = Rarely (It has occurred once)   2 = Sometimes (2-3 times)   3 = Many times (4-5 times)   4 = Very often (6 times or more)

1.	He/she has told me "I hate you!" "I wish you were dead!".	0	1	2	3	4
2.	He/she has insulted me.	0	1	2	3	4
3.	He/she has made offensive, degrading, and humiliating comments to me.	0	1	2	3	4
4.	He/she has threatened me (e.g. with hurting me, with hurting himself/herself, with running away from home).	0	1	2	3	4
5.	We watch what he/she wants on TV at home.	0	1	2	3	4
6.	He/she has demanded me to buy him/her things even knowing I can't afford them.	0	1	2	3	4
7.	He/she has incurred debts that I have had to pay.	0	1	2	3	4
8.	He/she has thrown things at me.	0	1	2	3	4
9.	When we have an argument he/she has the last word.	0	1	2	3	4
10.	He/she has hit me with something that could hurt me.	0	1	2	3	4
11.	He/she has kicked, slapped, and/or punched me.	0	1	2	3	4
12.	He/she has stolen money from me.	0	1	2	3	4
13.	He/she has told me that at home I have to do what he/she wants.	0	1	2	3	4
14.	He/she has demanded me to stop what I am doing to pay attention to him/her.	0	1	2	3	4

If your son/daughter has carried out any of the aforementioned behaviours, please indicate the frequency of **the reasons** for behaving in that way.

0: Never   1: Sometimes   2: Almost always   3: Always

1. Because he/she wants to get come home later after going out at night.	0	1	2	3
2. To get more money from me.	0	1	2	3
3. To be bought something he/she wants.	0	1	2	3
4. To avoid doing some chore (e.g., cleaning up her/his room).	0	1	2	3
5. To avoid going to school and/or studying.	0	1	2	3
6. Because of her/his own temper.	0	1	2	3
7. In response to a previous physical aggression from me (e.g., slap, punch, shove...).	0	1	2	3
8. In response to a previous verbal aggression from me (e.g., insult).	0	1	2	3

Cuestionario de Violencia Filio-parental Versión Padres (C-VIFIP-P) (Spanish)

Por favor, indique la frecuencia con la que su hij/a ha llevado a cabo cada una de las siguientes conductas durante el ultimo año.

0 = Nunca 1 = Raramente (ha ocurrido alguna vez) 2 = Algunas veces (2-3 veces) 3 = Bastantes veces (4-5 veces) 4 = Muy a menudo (6 veces o más)

1. Me ha llegado a decir cosas como “te odio”, “ojalá te mueras”.	0	1	2	3	4
2. Me ha insultado.	0	1	2	3	4
3. Me ha hecho comentarios ofensivos, negativos y/o degradantes.	0	1	2	3	4
4. Me ha amenazado (con hacerme daño, con hacerse daño a sí mismo/a, con irse de casa).	0	1	2	3	4
5. En casa se ve en la televisión lo que él/ella quiere.	0	1	2	3	4
6. Me ha exigido que le compre cosas incluso sabiendo que no me lo puedo permitir.	0	1	2	3	4
7. Ha adquirido deudas que yo he tenido que pagar.	0	1	2	3	4
8. Me ha lanzado cosas.	0	1	2	3	4
9. Al discutir conmigo, él/ella tiene la última palabra.	0	1	2	3	4
10. Me ha golpeado con algo que puede hacerme daño.	0	1	2	3	4
11. Me ha dado una patada, bofetada y/o puñetazo.	0	1	2	3	4
12. Me ha robado dinero.	0	1	2	3	4
13. Me ha exigido que en casa se haga lo que él/ella quiera.	0	1	2	3	4
14. Me ha exigido que deje lo que estoy haciendo para que le atienda.	0	1	2	3	4

Si su hijo/a ha mostrado alguna de las conductas anteriores, por favor, señale la frecuencia de las **razones** por las que cree que ha actuado de ese modo.

0: Nunca 1: Algunas veces 2: Casi siempre 3: Siempre

1. Por querer llegar más tarde a casa cuando sale por la noche.	0	1	2	3
2. Para que le dé más dinero.	0	1	2	3
3. Para que le compre algo que quiere.	0	1	2	3
4. Para evitar hacer alguna tarea (recoger su habitación, ayudar en las tareas de casa…).	0	1	2	3
5. Para evitar ir a clase, estudiar.	0	1	2	3
6. Por su propio carácter.	0	1	2	3
7. En respuesta a una agresión física mía (bofetada, puñetazo, empujón…).	0	1	2	3
8. En respuesta a una agresión verbal mía (por ejemplo insulto).	0	1	2	3
